# Phages Bearing Affinity Peptides to Bovine Rotavirus Differentiate the Virus from Other Viruses

**DOI:** 10.1371/journal.pone.0028667

**Published:** 2011-12-06

**Authors:** Xin Wang, Guangxing Li, Yudong Ren, Xiaofeng Ren

**Affiliations:** 1 Department of Preventive Veterinary Medicine, College of Veterinary Medicine, Northeast Agricultural University, Xiangfang District, Harbin, China; 2 Department of Preventive Veterinary Medicine, College of Veterinary Medicine, Northeast Agricultural University, Xiangfang District, Harbin, China; 3 Department of Computer, College of Engineering, Northeast Agricultural University, Xiangfang District, Harbin, China; Texas A&M, United States of America

## Abstract

The aim of this study was to identify potential ligands and develop a novel diagnostic test to pathogenic bovine rotavirus (BRV) using phage display technology. The viruses were used as an immobilized target followed by incubation with a 12-mer phage display random peptide library. After five rounds of biopanning, phages had a specific binding activity to BRV were isolated. DNA sequencing indicated that phage displayed peptides HVHPPLRPHSDK, HATNHLPTPHNR or YPTHHAHTTPVR were potential ligands to BRV. Using the specific peptide-expressing phages, we developed a phage-based ELISA to differentiate BRV from other viruses. Compared with quantitative real-time PCR (qPCR), the phage-mediated ELISA was more suitable for the capture of BRV and the detection limitation of this approach was 0.1 µg/ml of samples. The high sensitivity, specificity and low cross-reactivity for the phage-based ELISA were confirmed in receiver operating characteristics (ROC) analysis.

## Introduction

Neonatal calf diarrhea (NCD) is a common gastroenteritis infection worldwide, threatening the cattle production and causing substantial economic losses. Group A bovine rotavirus (BRV) is a causative agent of NCD [1]. Rotavirus belongs to the family of *Reoviridae* and is a non-enveloped virus; its genome is enclosed in three concentric layers and is composed of 11 segments of double-stranded RNA, which encode six structural proteins (VP1 to 4 and VP6 to 7) and five nonstructural proteins (NSP1 to 5) [2], [3]. According to the antigenic and genetic variations in the VP6 region, rotavirus can be divided into seven groups designated A–G. Group A BRV, the major viral pathogen for NCD can further be differentiated into 23 G- (glycoprotein) and 32 P- (protease sensitive protein) types, based on the VP7 and VP4 antigens, respectively [4]. Sporadic outbreaks of BRV in China have been reported recently [5]. Although the clinical course following rotavirus infection is typically short, virus can be detected in faeces for up to 3 weeks post-infection [6]. Some diagnostic procedures such as viral culture, RT-PCR and serology have been proved to be useful for detection of the pathogens, nevertheless, the World Health Organization recommends the use of enzyme immunoassays for the diagnosis of rotavirus infections [7].

Phage random peptide library is composed of a pool of billions of heterologous peptides expressed in the N terminus of the capsid protein of filamentous bacteriophages [8]. Phage random peptide library based phage display is well-developed technology to identify specific ligands of a target protein by a biopanning process. This technology can be applied in many fields such as antibody engineering [9], drug discovery and manufacture [10], pathogen diagnosis [11] and immunogen development [12].

The current study was initiated to identify potential ligands to BRV using phage display technology. By utilizing the phages bearing the ligands, we established a phage-based ELISA. The specificity, feasibility and suitability of the novel approach were compared to a commonly used quantitative real-time PCR (qPCR) in the context of differentiation of BRV from other pathogens.

## Materials and Methods

### Cell and virus

Monkey kidney epithelial (MA104) cells were grown in Dulbecco's MEM with 10% fetal bovine serum at 37°C. BRV strain HQ09 were propagated in the MA104 cells in the presence of trypsin (final concentration was 10 µg/mL) and purified by differential centrifugation conventionally. The protein concentration diluted in PBS was measured by Thermo Scientific NANODROP 2000 Spectrophotometer ((NanoDrop Technologies, Thermo Fisher Scientific, Wilmington, DE) and calculated by the absorbance ratio A_260_/A_280_ according to the manufacturer's instructions.

### Biopanning process

Phage display was performed based on the instructions of the reagent kit manufacturer (New England Biolabs) with minor modifications. For the first round of panning, 96-well plates were coated with the BRV at a concentration of 16 µg/well in 0.1 M NaHCO_3_ (pH 8.6) buffer overnight at 4°C. Then, the plates were blocked for 1 h at 4°C with 5% skimmed milk diluted in 0.05% (vol/vol) Tween 20 in phosphate-buffered saline (PBST). Following six washes with TBST (50 mM Tris-HCl, pH 7.5, 150 mM NaCl, 0.1%[vol/vol] Tween 20), the viruses were incubated with the phage library at a final concentration of 2×10^11^ (100 µl/well) at room temperature for 30 min with gentle rocking. The unbound phages were removed by 10 times wash with TBST and the bound phages were eluted by adding 100 µL elution buffer (0.2 M glycine-HCl [pH 2.2]) at room temperature for 30 min. The eluate neutralized with 15 µL 1 M Tris-HCl (pH 9.1) was harvested followed by amplification and titration in *Escherichia coli* ER2738.

The second and third rounds of panning were done by similar panning processes with the exception of gradually increased concentration of Tween 20 (0.5% [vol/vol]) in TBST. In the fourth round of panning, the coated viruses were replaced by the supernatant of MA104 culture. The phages were incubated with the supernatant at room temperature for 30 min prior to the fifth round of panning. The titer of the phages in input, elute buffer (output) and that after amplification in *E.coli* were analyzed according to the manufacturer's instructions.

### Binding activity of individual phage to BRV

Positive phage clones were identified by indirect ELISA. Briefly, ELISA plates were coated with BRV diluted in 0.1 M NaHCO_3_ (pH8.6) at a concentration of 10 µg/ml overnight at 4°C. Then, the plates were blocked with 1% bovine serum albumin (BSA) in TBS buffer (TBSB) at room temperature for 2 h followed by six times washes with TBST. The individual phages from the last round of biopanning at a concentration of 2×10^11^ in 0.1 M NaHCO_3_ (pH 8.6) were incubated with the coating viruses for 1 h at 37°C. The M13 polyclonal antibody (dilution 1∶1,000 in TBSB; Abcam) was added to these wells for 1 h at 37°C, after six washes with TBST. Subsequently, the horseradish peroxidase (HRP)-conjugated anti-rabbit IgG antibody (dilution 1∶5,000 in TBSB, Sigma) was added and the color development was done by using *o*-phenylenediamine (OPD) as substrate. The OD_492_ value was read using an ELISA plate reader. The experiments were performed in triplicate.

### Identification of phage displayed peptide sequences by PCR

Twelve positive phage clones were selected and amplified in *E.coli* followed by precipitation with polyethylene glycol-NaCl. The genome DNA of the phages was extracted and purified using a plasmid extraction kit (Qiagen, Germany). The gene encoding the exogenous peptides of M13 was amplified by PCR using the purified DNA template, sense primers: 5′-TCACCTCGAAAGCAAGCTGA and antisense primer: 5′-CCCTCATAGTTAGCGTAACG. The PCR parameters were composed of 95°C for 5 min, 30 cycles of 95°C for 30 s, 57°C for 30 s, 72°C for 30 s, together with a final extension at 72°C for 7 min. The encoding amino acid sequences were deduced post-sequencing.

### Establishment of phage-mediated ELISA for virus diagnosis

The selected phages were used as diagnostic reagents to detect a panel of viruses that was composed of bovine rotavirus (BRV), bovine herpesvirus type 1 (BHV-1), bovine viral diarrhea virus (BVDV), bovine coronavirus (BCV), porcine rotavirus (PRV), porcine transmissible gastroenteritis virus (TGEV), porcine epidemic diarrhea virus (PEDV), porcine reproductive and respiratory syndrome virus (PRRSV). All the viruses were diluted in 0.1 M NaHCO_3_ (pH8.6) to a final concentration of 1 µg/ml and coated onto ELISA plates overnight at 4°C followed by ELISA as above. The OD_492_ values were determined. At least three independent experiments were repeated. Statistical significance was evaluated using the t-test. The p<0.01 and was considered statistically highly significant.

### Determination of negative-positive cutoff

The negative-positive cutoff value was set by the average OD ratio of 100 field negative samples and 100 positive samples by phage-based ELISA. A negative-positive threshold for each assay was determined using the Microsoft Excel spreadsheet which was generated.

### Assay repeatability analysis

Twenty negative samples and twenty positive samples were included in the repeatability analysis. For intra-assay (within-plate) repeatability, three replicates of the same sample were performed in the same plate. For interassay (between-run) repeatability, three replicates of each sample were run in different plates on different occasions. Mean OD ratio; standard deviation (SD), and coefficient of variation (CV) of three replicates of each test were analyzed.

### Evaluation of correlation between phage–based ELISA and qPCR

The sensitivity of phage–based ELISA was compared with qPCR to determine the minimum quantity for the virus detection. Briefly, BRV at a concentration of 1 mg/ml was 10-fold serially diluted from 10^−1^ to 10^−6^ in DMEM. One aliquot of each dilution was used for the phage-based ELISA assay, and the another aliquot was used for qPCR. For phage-based ELISA, the BRV were coated into ELISA plates overnight at 4°C. Then, the wells were blocked with 5% skimmed milk for 4 h at room temperature. Thereafter, the selected phages and phage complex from the phage display library (control phage) diluted in PBS at a final concentration of 1.5×10^12^ was used as primary antibody and added onto the wells. After triple washes with TBST, the wells were sequentially incubated with anti-M13 antibody (1∶2000 dilution in PBS) for 1 h and HRP-conjugated goat anti-rabbit antibody (1∶5000 dilution in PBS) for 1 h. The OD_492_ value of detected phage wells >0.15 was judged as positive results.

The genome equivalents (GE) of BRV were determined by a qPCR assay using sense primer (P1: 5′- GATGGAGCGACTACATGGTATTTTAAT) and antisense primer (P2: 5′- TGGCTGTGCATTTGGAAATAAT), which was designed to amplify VP6 gene (216 bp in length) of BRV. Sense primer 5′- GTATCCTGACTCTCAAGTACCCCATT and antisense primer 5′- ATGGCCGGAACGTTGAAG) was used to amplify the housekeeping gene, beta-actin (208 bp in length) used as an internal reference. A volume of 100 µl of each sample's total RNA or RNA was extracted with a commercially available kit (HaiGene, China) according to the manufacturer's instructions. For reverse transcription, 5 µl of RNA (1 µg), 1 µl of primer P2 (10 pmol), 4 µl of 5×RT-PCR buffer (TaKaRa, China), 1 µl of M-Mulu (TaKaRa), 0.5 µl of RNase inhibitor, 2 µl dNTP (10 mM/each) and 7.5 µl of sterile water were gently mixed and incubated at 30°C for 10 min, 42°C for 1 h, 95°C for 5 min before cooling to 4°C and storage at −20°C. Subsequent real-time PCR was performed using ABI PRISM 7500 real-time PCR machine (Applied Biosystems, USA). The real-time PCR mixture included 1 µl of cDNA template (1 µg), 25 µl of SYBR Green PCR Mix, 1 µl of SYBR Taq polymerase, 1 µl of ROX Reference Dye, 1 µl of sense primer, 1 µl of antisense primer and 20 µl of sterile water. The PCR parameters was composed of 95°C for 2 min; 40 cycles of 95°C for 10 s and 60°C for 35 s. The expression level of BRV VP6 was normalized to that of beta-actin according to the comparative cycle threshold (CT) method used for quantification recommended by the manufacturer's protocol [13]. Delta CT was determined and each sample was analyzed in duplicate in the qPCR. The GE was determined using a standard curve; the mean and standard deviation were calculated. The detection limit was calculated to be 10 cDNA copies per reaction.

### Statistical analysis

Receiver operating characteristic (ROC) analysis and Spearman test were run using SPSS 17.0 to analyze the correlation between GE in qPCR and corresponding OD values in the phage-based ELISA.

## Results and Discussion

### Biopanning to identify phages bearing specific peptides to bovine rotavirus

In the past, immunoglobulins have been used to treat rotavirus diarrhea, nevertheless, application of immunoglobulins is relatively expensive and their side-effects are uncertain [14]. It is also reported that compounds isolated from the roots of G. uralensis may be potent anti-rotavirus agents *in vivo* [15], however, their antiviral effects have not been evaluated clinically. In vaccination, although progress has been made in the development of live, attenuated oral vaccines, improved vaccines are still needed, particularly in developing countries due to the unsatisfactory effect of those vaccines [16]. At present, effective diagnostic tests for screening viruses are important for prevention of BRV.

**Figure 1:**
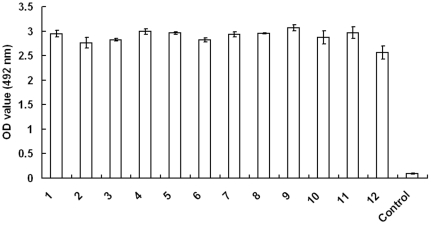
Binding analysis of the selected phages to BRV in ELISA. Twelve selected phages named phages 1 to 12 were incubated with the BRV in ELISA plates to test their binding activities to the viruses as described in Materials and methods. The individual phage and control are indicated in the x axis. The control is phage complex from the phage library. The OD_492_ values of the tested individual phages are shown on the y axis. Bars show the standard deviation from three independent assays.

Phage display technology is a good approach for ligand identification of targets and phage-displayed peptides from combinatorial libraries that interacting with hepatitis B virus, adenovirus type 2, Andes virus, Sin Nombre virus, Hantaan virus, Avian H5N1 Virus and coronavirus have been selected [17]–[21]. In this study, we used the BRV as an immobilized target followed by biopanning process using a 12-mer phage display peptide library to select ligands to this virus. Because the viruses were harvested from the cells, we performed a subtract panning to decrease potential reaction background. Additionally, we gradually increased concentration of Tween 20 to improve the specificity of biopanning. The results showed that the titer of the eluted phages was increased gradually (data not shown). After the last panning, twelve phage clones that were able to bind to BRV were identified by ELISA, in contrast, the control phages had a negligible binding ability to this virus (Figure 1).

### Peptide sequences displayed on the phages to BRV

After amplification of the selected phages, their genomes were extracted and the genes encoding the peptides expressed on the surfaces of the recombinant phages were amplified by PCR. Three deduced peptide sequences (12 amino acids in length) from the selected phages were identified (Table 1). Phages bearing peptides HVHPPLRPHSDK, YPTHHAHTTPVR or HATNHLPTPHNR were named phages HV, YP and HA, respectively. The sequence data has been deposited in the GenBank and their accession numbers will be provided once available. To our knowledge, this is the first report regarding the peptide sequences that can bind to the BRV. Further experiments will be focused on analyzing the role of the peptides/motifs in the infection mechanism of BRV.

**Table 1:** Deduced amino acid sequences of phage clones.

PCR product number	Sequences of deduced amino acids of each peptide
1	HVHPPLRPHSDK (HV)
2,3,4,5,7,8,9,10,11,12	YPTHHAHTTPVR (YP)
6	HATNHLPTPHNR (HA)

Twelve selected phages (phages 1 to 12) were subjected to DNA extraction and PCR. The deduced amino acid sequences are shown. Three peptide sequences identified are shown and designated as HV, YP and HA, respectively (in parenthesis).

### Phages expressing specific peptides differentiated BRV from other viruses

**Figure 2:**
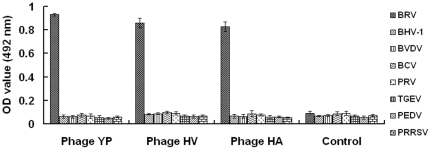
Phage-based ELISA differentiating BRV from other viruses. Three phages harboring specific peptides recognizing BRV were identified from the 12 selected phages. Three phages bearing different peptide sequences were identified and designated HV, YP and HA. Bovine rotavirus (BRV), bovine herpesvirus type 1 (BHV-1), bovine viral diarrhea virus (BVDV), bovine coronavirus (BCV), porcine rotavirus (PRV), porcine transmissible gastroenteritis virus (TGEV), porcine epidemic diarrhea virus (PEDV), porcine reproductive and respiratory syndrome virus (PRRSV) were coated onto ELISA plates followed by incubation with above-mentioned phages. Subsequent incubation included addition of anti-M13 antibody and HRP-conjugated antibody. The ratio between the OD_492_ value of individual phage and the OD_492_ value of the control phage complex from the phage library is shown on the y axis, respectively. “*” means p<0.01, compared with other groups. Bars show the standard deviation from three independent assays.

We then used the phages bearing the specific affinity peptides to BRV to develop a phage-mediated diagnostic assay to BRV. It is clearly important to distinguish BRV from other viruses that might cause multiple infection, therefore, the selected phages were analyzed for their specificities in recognizing BRV and a panel of selected viruses. As shown in Figure 2, the three identified phages were capable of recognizing BRV specifically rather than other control viruses (p<0.01); in contrast, the control phage complex from the phage library had a negligible reaction with these viruses used in this study. As amplification of phage is relatively cheap, the specific phages identified in this study may be specific and inexpensive assay reagents for detection of BRV. Further tests are needed to analyze the utility of the phage-mediated ELISA in detection of BRV from clinical samples.

### Confirmation of negative-positive cutoff

A cutoff point for each assay was determined so that DSN and DSP were maximized while the sum of false negative and false positive results was minimized. The OD at 492 nm for negative sample of YP, HV and HA ranged from 0.041 to 0.153. The averaged YP, HV and HA OD of 60 negative sample in the ELISA was 0.129611,0.12645 and 0.12784, yielding a suitable cut-off OD value of 0.150982,0.147325 and 0.148613 (mean+3SD) and indicated that 99% of the negative sample have YP, HV and HA OD values below 0.15. The positive threshold was set at 0.15 and all 60 positive samples had YP, HV and HA OD values above 0.15.

### Evaluation of assay repeatability

The repeatability test was done by comparing OD ratios of triplicate results from each field stool sample tested in the same plate (intra-assay repeatability) or in different plates at different times (inter-assay repeatability).The intra-assay CV of YP, HV and HA for 20 positive samples ranged from 0.68% to 2.79%, with a median value of 1.32%, 1.71% and 1.79%, while those of negative samples ranged from 4.57% to 21.94%, with a median value of 7.56%, 8.21% and 8.94%. The inter-assay CV of YP, HV and HA for positive samples was between 0.31% and 2.01%, with a median value of 1.24%, 1.41% and 1.79%, whereas the CV for negative samples was between 1.37% and 6.74%, with a median value of 2.25%, 3.07% and 2.82%. These data showed that the newly developed phage-based ELISA was repeatable and had a relatively low variation.

### Evaluation of correlation between phage–based ELISA and qPCR

**Figure 3:**
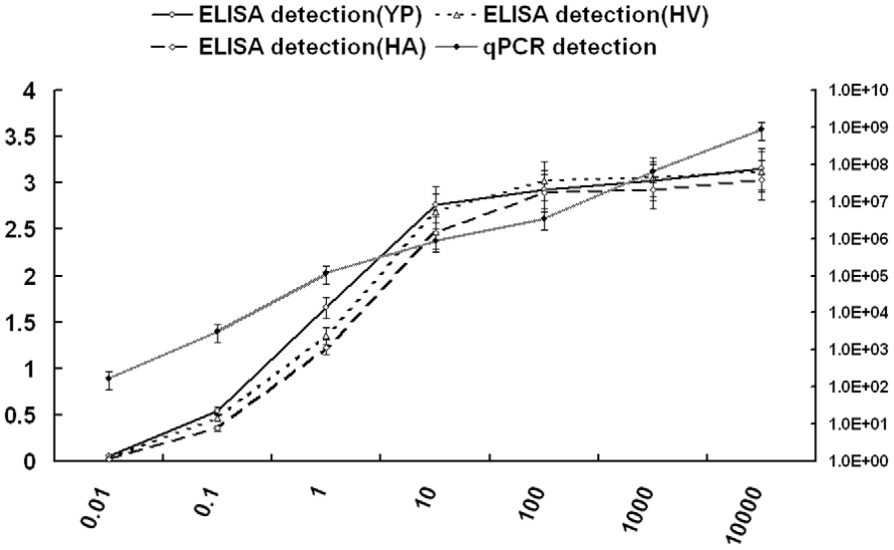
Detection of serial dilutions of purified BRV using phage-mediated ELISA and qPCR. Serially diluted BRV was used as coating antigen followed by successive incubation with phages YP, HV or HA, anti-M13 antibody and HRP-conjugated goat anti-rabbit antibody. The OD_492_ of detected phage wells is shown in the left y axis. Viral RNA was also extracted from the serially diluted viruses, subsequently, the cDNA was achieved by reverse transcription and the resulting cDNA was ten-fold serially diluted and subjected to qPCR. The DNA copies are shown in the right y axis. The concentration of the viruses is indicated in the x axis. The ELISA and qPCR assays were performed in triplicate. Bars show the standard deviation from three independent assays.

**Figure 4:**
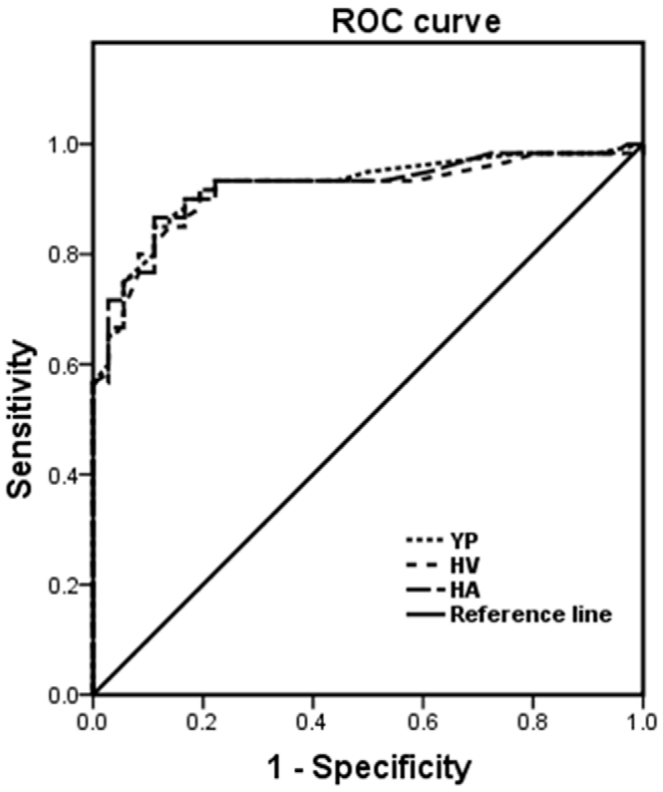
ROC curve for detection of BRV of the phage-mediated ELISA in comparison to the qPCR. The ROC (receiver operating characteristics) analysis for all samples to each phage detection (YP, HV or HA) was performed, respectively according to the area under the ROC curves (AUC). The results of the qPCR analysis of these samples were used as “gold standard” reference. The ROC plots the true positive rate (sensitivity) against the false positive rate (1-specificity). The diagonal indicates no discriminatory power.

To evaluate the diagnostic accuracy of the phage-mediated ELISA, the qPCR analysis on BRV detection was set as the reference for comparison of the phage–based ELISA results (Figure 3). Furthermore, the correlation between phage–based ELISA and qPCR was evaluated by determining the area under the ROC-curve (AUC). As shown in Figure 4, the ROC-analysis was very high and comparable AUC-values exceeding 0.9 using either the results of YP, HV or HA as one matrix (the ROC-curve of YP, HV and HA was 0.923, 0.914 and 0.921, respectively). As high values close to 1 of the ROC-curve represent a highly accurate test, the phage-mediated ELISA is a rather sensitive diagnostic approach for BRV. The dependency between GE of qPCR and OD extinction values of the established ELISA was analyzed using Spearman's rank test. For the Spearman test, Spearman's rho of YP, HV and HA was 0.710, 0.695 and 0.707, respectively (p<0.01). This result indicates that a stronger correlation between GE in qPCR and OD in phage–based ELISA. In conclusion, in this study, we for the first time identified the potential ligands to BRV; the phages bearing the peptides to BRV had potential as good diagnostic reagents for development of a novel and sensitive phage-mediated ELISA to detect BRV.
